# Navigating the Prognostic Complexity of IgH Cytogenetic Abnormalities in Multiple Myeloma Among Diverse IgH Cytogenetic Abnormalities–Involved High‐Risk Stratifications

**DOI:** 10.1002/cam4.71152

**Published:** 2025-08-27

**Authors:** Yanqiu Xiong, Meng Li, Yan Li, Wenjiao Tang, Tian Dong, Bing Xiang, Li Zhang, Ling Pan, Ting Niu

**Affiliations:** ^1^ Department of Hematology, Institute of Hematology, West China Hospital, Sichuan University Chengdu Sichuan China; ^2^ Department of Hematology Clinical Medical College, Affiliated Hospital of Chengdu University, Chengdu University Chengdu Sichuan China

**Keywords:** chromosome 14, cytogenetic abnormality, IgH, myeloma, prognosis, transplant

## Abstract

**Objective:**

To evaluate the clinical and prognostic significance of immunoglobulin heavy chain (IgH) cytogenetic abnormalities in patients with newly diagnosed multiple myeloma (NDMM) and explore their association with other high‐risk cytogenetic features.

**Patients and Methods:**

We retrospectively analyzed 503 NDMM patients treated between February 1, 2016, and February 29, 2024. Cytogenetic abnormalities were identified by fluorescence in situ hybridization.

**Results:**

The study uncovered a significant correlation between IgH cytogenetic abnormality and an increased prevalence of anemia (*p* < 0.001), thrombocytopenia (*p* = 0.005), and the presence of high‐risk cytogenetic aberrations, including +1q21 (*p* < 0.001) and P53 deletion (*p* = 0.001). Patients afflicted with IgH cytogenetic abnormality were found to have more advanced stages of the disease, as indicated by higher Disease Stage (*p* < 0.001), International Staging System (*p* = 0.018), Revised International Staging System (RISS) (*p* < 0.001), and Second Revision of the International Staging System (*p* < 0.001). This cytogenetic abnormality was also linked to a markedly diminished progression‐free survival (PFS) across RISS I–II, yet autologous stem cell transplantation (ASCT) offered significant improvement for PFS (*p* < 0.05). Notably, the specific t(14;16) and t(14; undefined) were significantly associated with shorter PFS (*p* < 0.001) and shorter overall survival (OS) (*p* < 0.05). Additionally, the confluence of IgH cytogenetic abnormality with other cytogenetic abnormalities, particularly +1q21, P53 deletion, RB/D13S319 deletion, and elevated LDH level, was found to exacerbate the disease outcome.

**Conclusion:**

IgH cytogenetic abnormalities indicate aggressive disease and poorer survival in NDMM, especially when accompanied by other high‐risk markers. ASCT may mitigate these adverse outcomes, supporting its role in individualized treatment strategies.

## 1 Introduction

The immunoglobulin heavy chain (IgH) cytogenetic abnormality on chromosome 14 is a common genetic abnormality in multiple myeloma (MM) [1]. It is thought that MM initiates through chromosomal cytogenetic abnormalities involving an IgH locus or hyper‐diploid (HDR) [2]. IgH enhancer activity is extremely high in plasma cells, enabling the translation of large amounts of immunoglobulin. IgH cytogenetic abnormalities result in strong expression of oncogenes located near the IgH enhancer. Primary IgH translocations include t(14;16), t(14;20), t(11;14), t(4;14), and t(6;14), which respectively result in MAF, MAFB, CCND1, MMSET/FGFR3, and CCND3 being ectopically overexpressed [3].

The heterogeneity of chromosomal abnormalities, particularly the specific forms of IgH cytogenetic abnormalities in MM, poses a significant challenge in defining high‐risk criteria across various prognostic standards. The cytogenetic features of high‐risk MM include t(4;14), TP53 mutation, t(14;20), deletion 17p, t(14;16), or gain/amplification of 1q [4]. Double‐hit and triple‐hit MM respectively indicate that there are two high‐risk features present and three or more high‐risk features present [5]. In Mayo Stratification of Myeloma and Risk‐Adapted Therapy (mSMART) additional cytogenetics and other markers are incorporated to assess risk [4]. The high‐risk category includes gain/amplification of 1q, t(14;16), TP53 mutation, t(4;14), del(17p), or t(14;20) as identified by fluorescence in situ hybridization (FISH). Furthermore, the guidelines established by the National Comprehensive Cancer Network (NCCN) for MM indicate that genetic high‐risk characteristics include gain/amplification of 1q21, alongside the established abnormalities of t(14;16), del(17p), and t(4;14) [6].

This study aims to elucidate the prognostic significance of IgH cytogenetic abnormalities in newly diagnosed MM (NDMM), particularly focusing on the genomic abnormalities linked to a poor prognosis and the various criteria used to define high‐risk IgH translocations. This paper explores the nuances of high‐risk criteria and their impact on patient outcomes in order to provide a more comprehensive understanding of the role of IgH cytogenetic abnormalities in MM prognosis.

## 2 Methods

### 2.1 Patients

This study cohort included 503 NDMM patients monitored at West China Hospital, Sichuan University from February 2016 to February 2024, with cytogenetic abnormalities or no lesions by FISH. All patients were diagnosed and treated according to the International Myeloma Working Group (IMWG) criteria [7], with Durie–Salmon staging (DS) score, International Staging System (ISS) score, Revised International Staging System (RISS) score, and Second Revised International Staging System (R2ISS) score being calculated [8]. The majority of treatments were administered prior to the widespread clinical use of CD38 monoclonal antibodies. Induction schemes included proteasome inhibitor‐based regimens (e.g., bortezomib‐dexamethasone [BD], bortezomib‐cyclophosphamide‐dexamethasone [BCD]), immunomodulator‐based regimens (e.g., lenalidomide‐dexamethasone [RD], bortezomib‐lenalidomide‐dexamethasone [BRD]), and other treatment options (e.g., cyclophosphamide‐dexamethasone [CD]). This study aimed to evaluate the impact of various treatment modalities across various cytogenetic subtypes. The analysis was confined to patients who had documented first‐ and second‐line treatments, as delineated by IMWG consensus criteria [9]. Extramedullary disease (EMD) refers to the situation where myeloma cells can colonize and grow outside the bone marrow in certain multiple myeloma patients. Based on the location of the tumor, EMD can be categorized into two types: extramedullary bone related and extramedullary extraosseous. This study was approved by the Ethics Committee of West China Hospital in accordance with the Declaration of Helsinki (Approval number: 2024‐1889). All participants signed informed consent.

### 2.2 Chromosomal Abnormalities (CAs)

Bone marrow aspirates anticoagulated with heparin were collected, and bone marrow mononuclear cells (BMMCs) were subsequently isolated utilizing the Ficoll density gradient centrifugation method. The BMMCs were cultured for approximately 24–72 h, after which colchicine was added to arrest mitosis. The cells were then subjected to hypotonic treatment, fixation, aging, and G‐banding for karyotype analysis.

### 2.3 FISH Testing

Bone marrow samples were obtained following anticoagulation with heparin. CD138+ plasma cells were isolated using magnetic bead separation with a median purity of 80%, and a minimum of 1 × 10^6^ cells were processed per sample. FISH was then conducted on the purified plasma cells, examining a total of 400 interphase nuclei. The FISH panel included the following probes: IgH/FGFR3, IgH/MAFB, IgH/CCND3, IgH/CCND1, IgH/MAF, 1q21, 1p32, p53, D13S319, and RB1. Additionally, the study specifically focused on the significance of IgH cytogenetic abnormalities in MM. We calculated the mean value plus three standard deviations from 20 normal controls as the technical cutoff value within our center. The thresholds for IgH translocation were as follows: IgH/FGFR3 (3.33%), IgH/CCND1 (2.94%), IgH/MAFB (3.5%), IgH/CCND3 (2.58%), and IgH/MAF (3.48%).

### 2.4 Statistical Analysis

Progression‐free survival (PFS) was defined as the duration from the date of diagnosis to the occurrence of death, disease progression, relapse, or the last follow‐up, whichever event transpired first. Overall survival (OS) was measured as the interval between diagnosis and death or the last follow‐up, in accordance with international uniform response criteria [10]. Categorical variables across various groups were analyzed utilizing the chi‐square test, with Fisher's exact test being applied when deemed appropriate. PFS and OS were estimated using the Kaplan–Meier method. Survival curve comparisons were conducted utilizing the log‐rank test and a proportional hazards Cox regression model. The outcomes of the Cox models were expressed as hazard ratios (HR) accompanied by 95% confidence intervals (95% CI). Statistical analyses were executed using SPSS version 26.0 and Prism version 9.0 software.

## 3 Results

### 3.1 Higher Sensitivity of FISH Compared to Chromosome Banding Analysis in Detecting IgH Cytogenetic Abnormality in NDMM

In this study, among 503 patients with NDMM, 169 cases (33.60%) were detected with IgH cytogenetic abnormality using FISH, including the t(4;14) subgroup (*n* = 55, 10.93%), the t(11;14) subgroup (*n* = 33, 6.56%), the t(6;14) subgroup (*n* = 8, 1.59%), and the t(14;16) subgroup (*n* = 6, 1.19%). There were no cases with t(14;20). Additionally, 69 cases (13.72%) of patients showed cytogenetic abnormalities involving the 14q32 locus, with partner genes that were undefined, denoted as t(14; undefined). Meanwhile, 447 patients underwent chromosome culture, and 4 cases (0.89%) of IgH translocation were detected, among which 1 case showed t(8;14), 1 case showed t(1;14), 1 case showed t(2;14), and 1 case showed t(6;14). Compared with chromosome culture, FISH had a higher sensitivity in detecting IgH cytogenetic abnormality (33.60% vs. 0.89%, *p* < 0.001).

**FIGURE 1:**
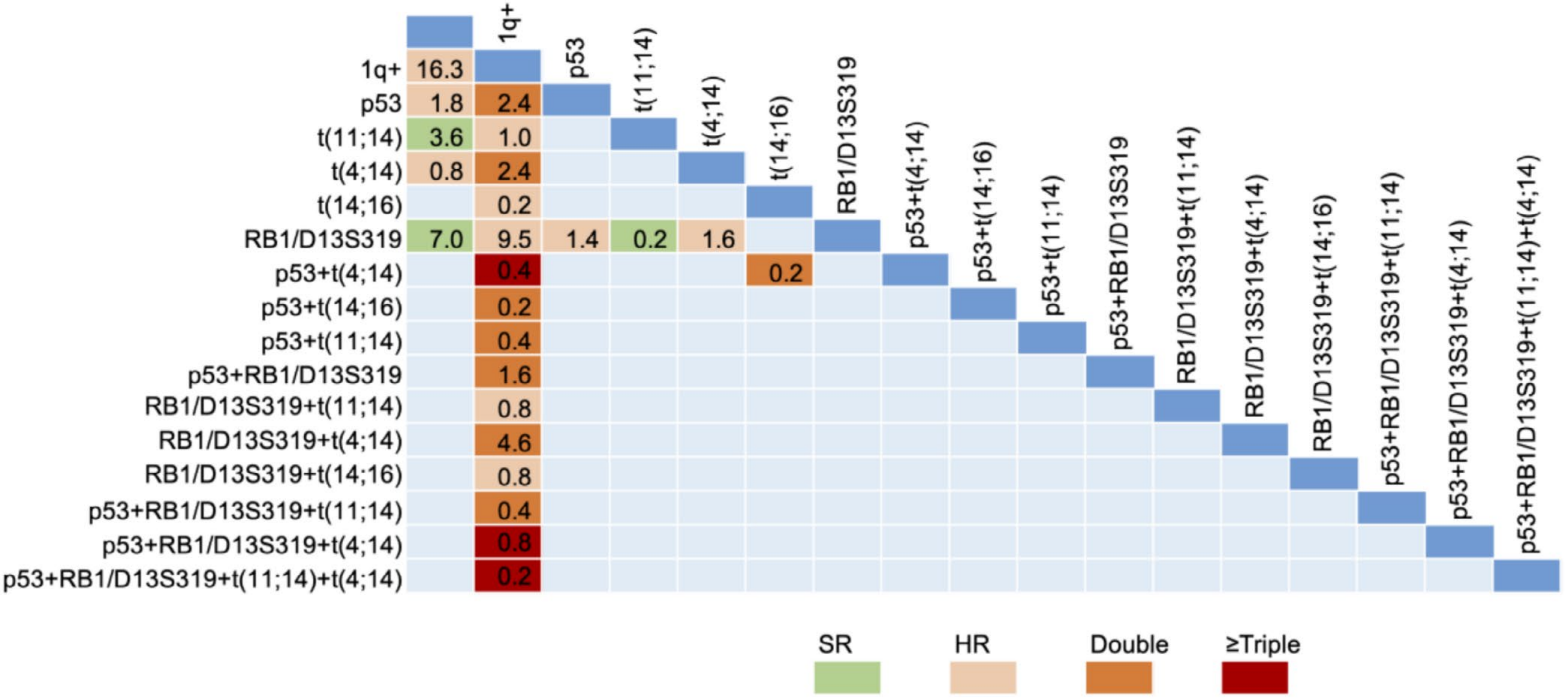
Constituents and distribution of cytogenetic abnormalities in 503 patients with NDMM. The numbers in the figure represent the percentages (%) of the corresponding cytogenetic abnormalities. double, double‐hit; HR, high‐risk; SR, standard‐risk; triple, triple‐hit.

Based on these findings, to ensure an adequate sample size for further analysis, the subsequent data in this study were based on FISH results. Among 503 patients with baseline results of six common cytogenetic abnormalities, the patients carrying 1, 2, 3, 4, and 5 cytogenetic abnormalities accounted for 29.4%, 18.7%, 8.9%, 1.2%, and 0.2%, respectively, as shown in Figure 1.

### 3.2 MM Exhibiting IgH Cytogenetic Abnormalities Showed Significantly Higher Incidences of Advanced Disease Stages, Severe Anemia, Thrombocytopenia, and a Higher Prevalence of Adverse Prognostic Genetic Abnormalities

When assessed against patients characterized as high‐risk (HR) without IgH translocations according to mSMART 3.0 criteria, those with HR IgH translocations were at a significantly greater risk of presenting with DS stage III (*p* = 0.002) and ISS stage III (*p* = 0.003). This trend was consistent with comparisons made based on NCCN guidelines, where the presence of HR IgH translocations was also linked to a higher likelihood of DS‐III (*p* = 0.002) and ISS‐III (*p* = 0.003), as detailed in Table 1.

**TABLE 1:** Clinical features of NDMM patients with IgH translocation involved HR.

Characteristics	mSMART3.0/NCCN			
	IgH translocation involved HR	No‐IgH translocation involved HR	OR (95% CI)	*p*
	*N* = 61 (12.13%)	*N* = 442 (87.87%)		
Age ≥ 65 years	26 (42.62%)	162 (36.65%)	1.284 (0.746–2.210)	0.366
Gender M/F	33 (54.09%)/28 (45.91%)	246 (55.65%)/196 (45.35%)	1.065 (0.622–1.823)	0.819
EMD	6 (9.84%)	74 (16.74%)	0.543 (0.225–1.306)	0.167
RI	22 (36.07%)	147 (33.26%)	1.132 (0.647–1.979)	0.663
β2M > 5.5 mg/L	22 (36.07%)	136 (30.77%)	1.263 (0.725–2.223)	0.403
High‐LDH	14 (22.95%)	101 (22.85%)	1.006 (0.532–1.901)	0.986
DS (III)	41 (67.21%)	203 (45.93%)	2.414 (1.370–4.252)	0.002
ISS (III)	34 (55.74%)	159 (35.97%)	2.241 (1.304–3.851)	0.003
ORR (*n* = 454)	40/54 (74.07%)	318/400 (79.50%)	0.737 (0.383–1.419)	0.359
PFS, months (Mean ± SD)	21.11 ± 17.17	25.45 ± 20.04	NA	0.108
OS, months (Mean ± SD)	30.51 ± 22.08	32.74 ± 22.99	NA	0.475

Abbreviations: β2M, β2‐microglobulin; DS, Durie–Salmon staging system; EMD, extramedullary disease; ISS, international staging system; LDH, lactic dehydrogenase; M/F, male/female; ORR, overall response rate; OS, overall survival; PFS, progression‐free survival; RI, renal impairment.

Furthermore, patients harboring IgH cytogenetic abnormalities displayed more severe clinical features compared to those without such cytogenetic abnormalities, as outlined in Table 2. This group experienced significantly severe anemia (*p* < 0.001), thrombocytopenia (*p* = 0.005) and higher β2M (*p* = 0.013). The presence of IgH cytogenetic abnormalities also correlated with a higher incidence of HR cytogenetic abnormalities, including +1q21 (*p* < 0.001) and P53 deletions (*p* < 0.001), along with an increased likelihood of concurrent deletions of RB1 and D13S319 (*p* < 0.001).

**TABLE 2:** Comparison of baseline characteristics between NDMM patients with IgH cytogenetic abnormality and without IgH cytogenetic abnormality.

Characteristics	IgH cytogenetic abnormality	No‐IgH cytogenetic abnormality	OR (95% CI)	*p*
	*N* = 169 (33.60%)	*N* = 334 (66.60%)		
Age ≥ 65 years	59 (34.91%)	130 (38.92%)	0.842 (0.573–1.237)	0.380
Gender M/F	98 (57.99%)/71 (42.01%)	179 (53.59%)/155 (46.41%)	0.837 (0.576–1.216)	0.349
EMD	21 (12.43%)	61 (18.26%)	0.635 (0.372–1.084)	0.094
RI	57 (33.73%)	112 (33.53%)	1.009 (0.682–1.492)	0.965
β2M > 5.5 mg/L	72 (42.60%)	105 (31.44%)	1.619 (1.104–2.373)	0.013
High‐LDH	42 (24.85%)	73 (21.86%)	1.182 (0.765–1.826)	0.450
Platelet, ×10^9^/L (Mean ± SD)	160.30 ± 71.79	178.91 ± 74.99	NA	0.005
Hemoglobin, g/L (Mean ± SD)	97.24 ± 27.08	103.24 ± 28.99	NA	< 0.001
DS (III)	133 (78.70%)	202 (60.48%)	2.414 (1.573–3.706)	< 0.001
ISS (III)	77 (45.56%)	116 (34.73%)	1.573 (1.079–2.294)	0.018
RISS (III)	56 (33.14%)	47 (14.07%)	3.026 (1.940–4.721)	< 0.001
R2ISS (IV)	47 (27.81%)	22 (6.59%)	5.463 (3.159–9.449)	< 0.001
Complex‐Karyotype abnormalities	17/153 (11.11%)	36/294 (12.24%)	0.896 (0.485–1.654)	0.725
P53 deletion	27 (15.98%)	22 (6.59%)	2.697 (1.485–4.889)	< 0.001
1q21 (Cns ≥ 3)	108 (63.91%)	103 (30.84%)	3.971 (2.687–5.867)	< 0.001
1q21 (Cns = 3)	61 (36.09%)	60 (17.96%)	NA	< 0.001
1q21 (Cns ≥ 4)	47 (27.81%)	42 (12.57%)		
RB (+)/D13S319 (+)	81 (47.93%)	65 (19.46%)	3.809 (2.539–5.714)	< 0.001

Abbreviations: β2M, β2‐microglobulin; DS, Durie–Salmon staging system; EMD, extramedullary disease; ISS, international staging system; LDH, lactic dehydrogenase; M/F, male/female; ORR, overall response rate; OS, overall survival; PFS, progression‐free survival; RB(+)/D13S319(+), the absence of RB and the absence of D13S319 exist together; RI, renal impairment; RISS, revised international staging system.

Compared with NDMM without IgH cytogenetic abnormality, those with IgH cytogenetic abnormality had a more advanced stage, among which were DS‐III (*p* < 0.001), ISS‐III (*p* = 0.018), RISS‐III (*p* < 0.001) and R2ISS‐IV (*p* < 0.001) (Table 2).

### 3.3 IgH Cytogenetic Abnormality Significantly Shortens PFS Across RISS I–II Stages in NDMM

**FIGURE 2:**
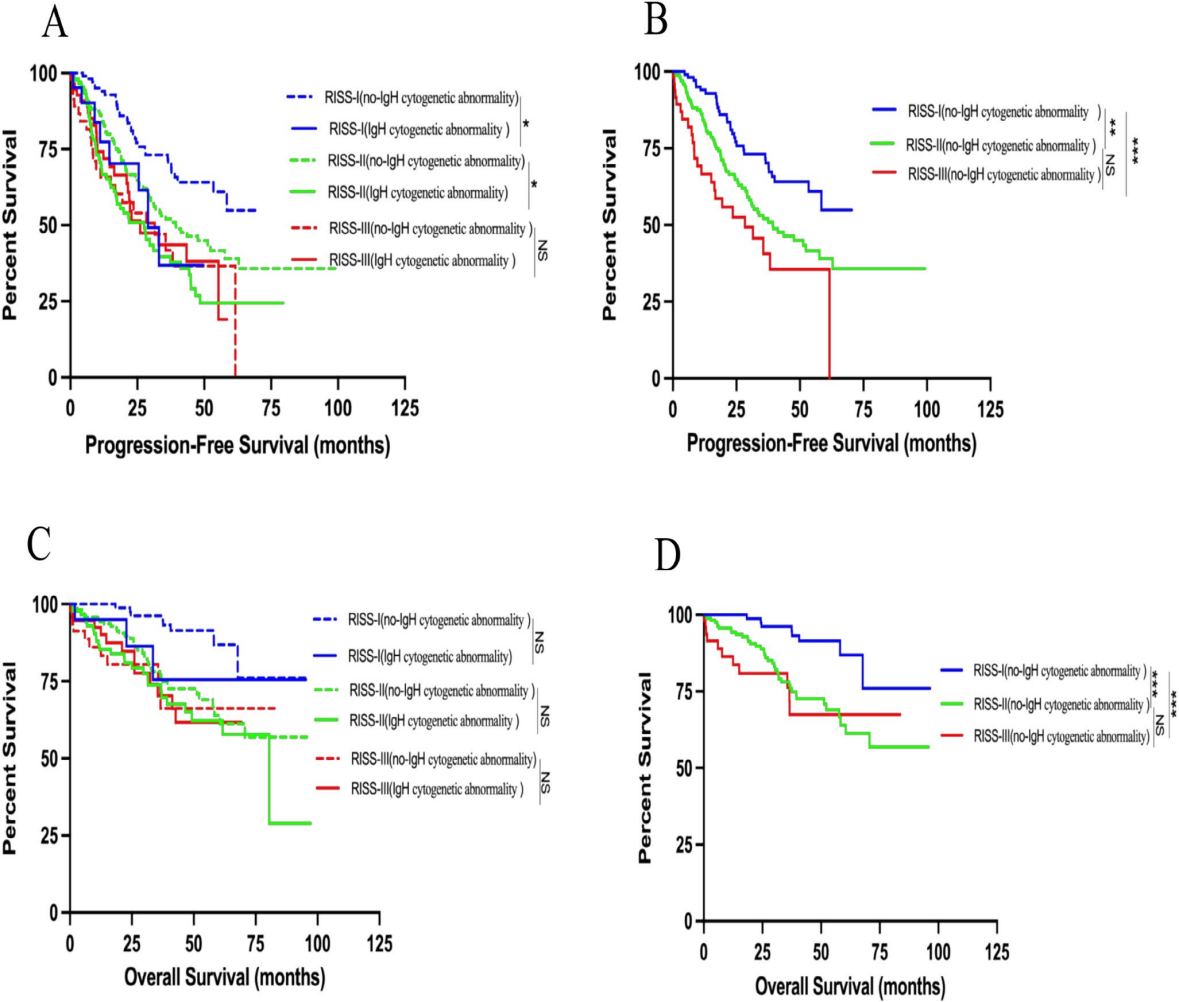
Impact of IgH cytogenetic abnormality on the outcomes of patients with NDMM in different RISS stratification. (A) Kaplan–Meier estimates of PFS in different RISS stratification without IgH cytogenetic abnormality; (B) Kaplan–Meier estimates of PFS with or without IgH cytogenetic abnormality in different RISS stratification; (C) Kaplan–Meier estimates of OS in different RISS stratification without IgH cytogenetic abnormality; (D) Kaplan–Meier estimates of OS with or without IgH cytogenetic abnormality in different RISS stratification; NS, not significant; ***p < 0.001, **p < 0.01, *p < 0.05, by two‐sided log‐rank test.

In the RISS staging, 26.84% of NDMM cases were in RISS‐I stage, 52.68% were in RISS‐II stage, and 20.48% were in RISS‐III stage. Without IgH cytogenetic abnormality, PFS in the RISS‐II group was significantly shorter than in RISS‐I (*p* = 0.003, HR = 1.849). PFS in RISS‐III stage was also shorter than in RISS‐I stage (*p* < 0.001, HR = 2.708), but there was no remarkable difference between RISS‐III and RISS‐II (*p* = 0.092, HR = 1.489) (Figure 2B). The RISS‐II stage had a significantly shorter OS than the RISS‐I stage (*p* < 0.001, HR = 3.510). Furthermore, OS of the RISS‐III stage was found to be significantly shorter compared to the RISS‐I stage (*p* < 0.001, HR = 4.548). In contrast, no statistically significant difference in OS was observed between the RISS‐III and RISS‐II stages (*p* = 0.576, HR = 1.211) (Figure 2D).

In the RISS‐I stage, the PFS of the cohort with IgH cytogenetic abnormality was significantly shorter than that of the cohort without IgH cytogenetic abnormality (*p* = 0.032, HR = 2.269) (Figure 2A). Similarly, in the RISS‐II stage, the PFS of the IgH cytogenetic abnormality cohort was also shorter compared to the cohort without IgH cytogenetic abnormality (*p* = 0.010, HR = 1.568) (Figure 2A). However, in the RISS‐III stage, there was no remarkable difference in PFS between the two groups (*p* = 0.869, HR = 0.954) (Figure 2A). Furthermore, no significant difference in OS was observed between the two groups across RISS stages I, II, and III, which were RISS‐I (with IgH cytogenetic abnormality) vs. RISS‐I (no‐IgH cytogenetic abnormality) (*p* = 0.099, HR = 2.899), RISS‐II (IgH cytogenetic abnormality) vs. RISS‐II (no‐IgH cytogenetic abnormality) (*p* = 0.218, HR = 1.365), RISS‐III (IgH cytogenetic abnormality) vs. RISS‐III (no‐IgH cytogenetic abnormality) (*p* = 0.945, HR = 1.029) (Figure 2C).

### 3.4 ASCT Overcame Poor Outcomes for MM With IgH Cytogenetic Abnormality

**FIGURE 3:**
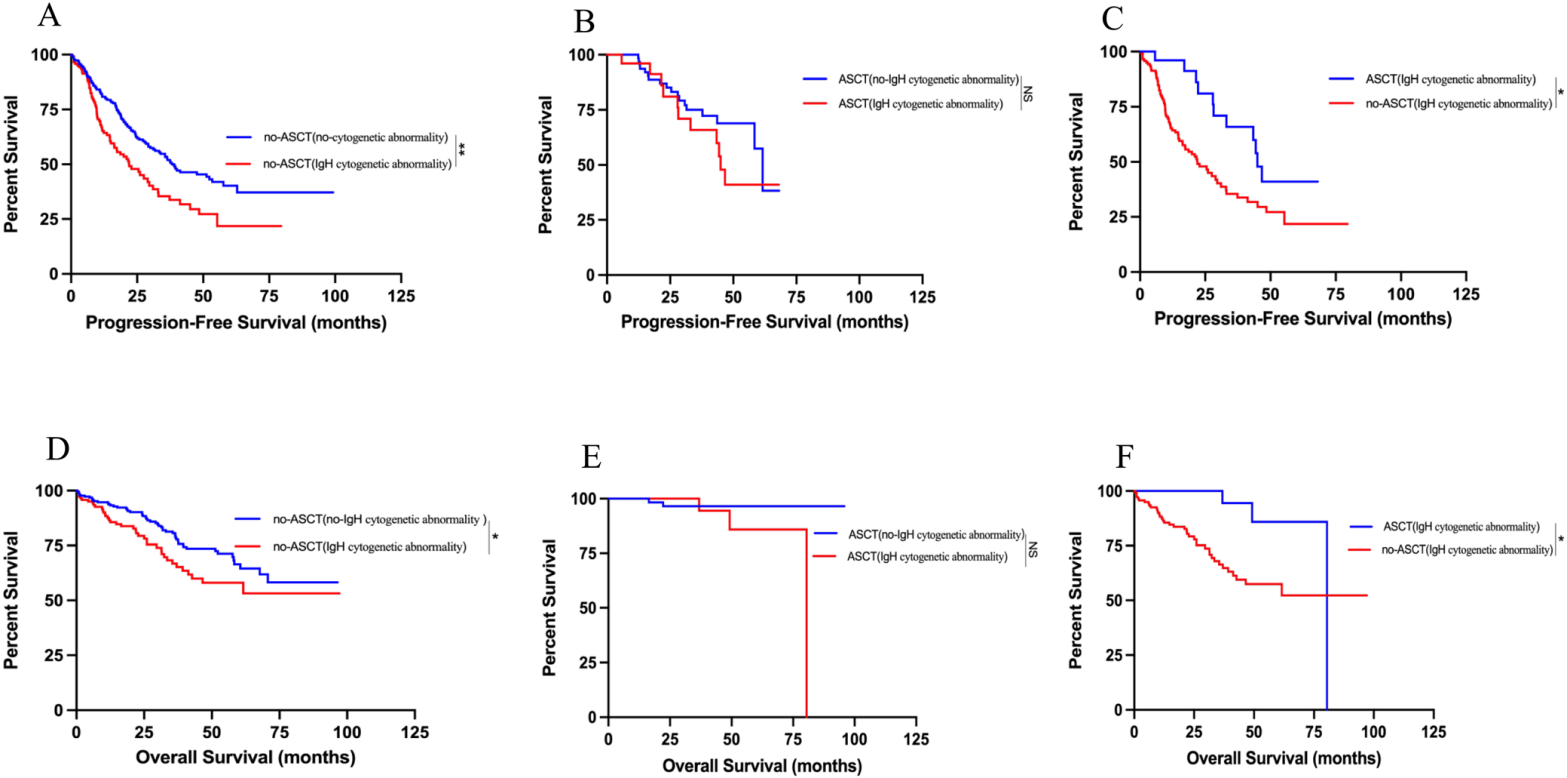
PFS and OS in 503 NDMM with or without IgH cytogenetic abnormality, considering ASCT status. (A) Kaplan–Meier estimates of PFS with or without IgH cytogenetic abnormality for no‐ASCT NDMM; (B) Kaplan–Meier estimates of PFS for patients with or without IgH cytogenetic abnormality for ASCT NDMM; (C) Kaplan–Meier estimates of PFS for IgH cytogenetic abnormality patients with or without ASCT; (D) Kaplan–Meier estimates of OS with or without IgH cytogenetic abnormality for no‐ASCT NDMM; (E) Kaplan–Meier estimates of OS with or without IgH cytogenetic abnormality for ASCT NDMM; (F) Kaplan–Meier estimates of OS for IgH cytogenetic abnormality patients with or without ASCT. ***p < 0.001, **p < 0.01, *p < 0.05, by two‐sided log‐rank test.

The median follow‐up time for this cohort of patients was 34.57 months, with a 95% confidence interval of 29.15–39.99 months. Patients with IgH cytogenetic abnormality exhibited a significant improvement in PFS, rising from 22.00 to 44.97 months (*p* = 0.013, HR = 2.166) after ASCT (Figure 3C).

Within the ASCT cohort, the groups with and without IgH cytogenetic abnormality showed no statistically significant difference in PFS as indicated by median values of 44.97 and 61.60 months, respectively (*p* = 0.258, HR = 1.535) (Figure 3B). Conversely, in the cohort without ASCT, individuals with IgH cytogenetic abnormality exhibited a notably shorter PFS in comparison to those lacking IgH cytogenetic abnormality, with respective median values of 22.00 and 38.20 months (*p* = 0.001, HR = 1.638) (Figure 3A).

Similarly, within the ASCT cohort, OS did not significantly differ between groups with and without IgH cytogenetic abnormality (80.46 months vs. NR, *p* = 0.144, HR = 3.487) (Figure 3E). However, in the no‐ASCT cohort, patients with IgH cytogenetic abnormality had shorter OS compared to those without (both NR, *p* = 0.024, HR = 1.596) (Figure 3D).

### 3.5 For NDMM With t(11;14) and Those Without IgH Cytogenetic Abnormality, There Were No Statistical Differences in PFS and OS

**FIGURE 4:**
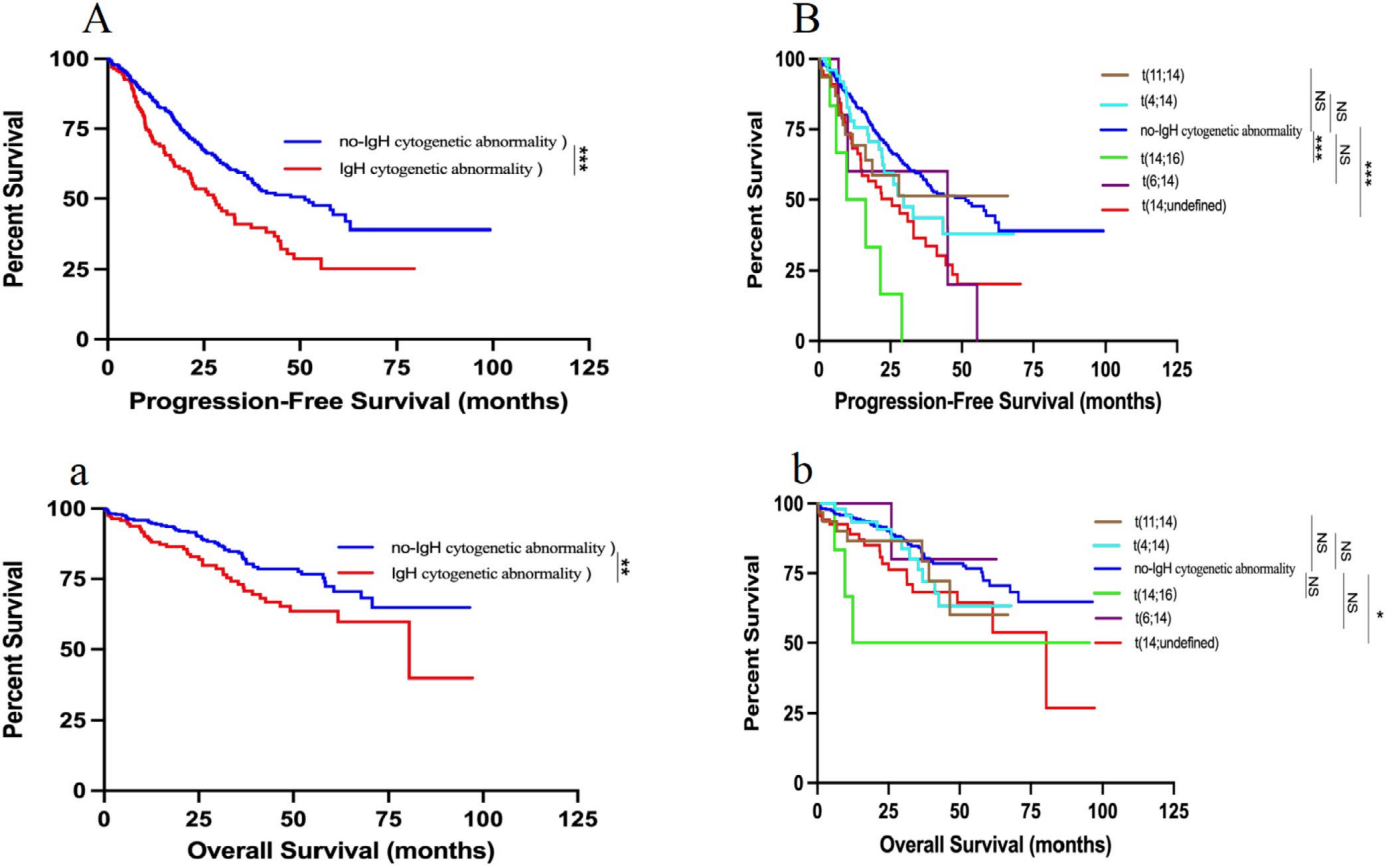
Subgroup analysis of IgH cytogenetic abnormality and different t(14) for PFS and OS in MM. (A) Kaplan–Meier estimates of PFS with or without IgH cytogenetic abnormality for 503 NDMM; (B) Kaplan–Meier estimates of PFS with different t(14) Subgroups for 503 NDMM; (a) Kaplan–Meier estimates of OS with or without IgH cytogenetic abnormality for 503 NDMM; (b) Kaplan–Meier estimates of OS with or without different t(14) Subgroups for 503 NDMM; ***p < 0.001, **p < 0.01, *p < 0.05, by two‐sided log‐rank test.

To investigate the clinical significance of different cytogenetic abnormalities involving chromosome 14, we conducted a subgroup analysis of various t(14) types. In this study, there was no statistically significant difference in PFS (*p* = 0.333, HR = 1.338, 95% CI: 0.685–2.613) and OS (*p* = 0.184, HR = 1.690, 95% CI: 0.638–4.480) between patients with t(11;14) and those without IgH cytogenetic abnormality (Figure 4B and Figure 4b). Therefore, we did not consider that t(11;14) had special clinical significance, and the data of the t(11;14) group were not presented separately in the subsequent part.

### 3.6 IgH Cytogenetic Abnormality Was a Prognostic Factor for PFS and OS in MM, but Not an Independent Predictor

The median OS and PFS of all patients were NR months and 39.00 (95% CI, 34.43–46.57) months, respectively. When comparing groups, the t(14;16) group exhibited a significantly shorter PFS compared to the no‐IgH cytogenetic abnormality group (*p* < 0.001, HR = 4.727, 95% CI: 0.849–26.330); similarly, the t(14; undefined) group showed a shorter PFS compared to the no‐IgH cytogenetic abnormality group (*p* < 0.001, HR = 1.963, 95% CI: 1.251–3.080) (Figure 4B). Additionally, the OS of the t(14; undefined) group shows a significant decrease relative to the group without IgH cytogenetic abnormality (*p* = 0.016, HR = 1.875, 95% CI: 1.000–3.513) (Figure 4b). However, when compared with the group lacking IgH cytogenetic abnormality, the presence of t(14;16) did not result in a statistically significant difference in OS between the groups (*p* = 0.106, HR = 2.516, 95% CI: 0.422–15.010) (Figure 4b).

An univariate analysis of a cohort of 503 NDMM patients was performed to determine the impact of extramedullary disease (EMD), age, DS stage, ISS stage, LDH level, renal insufficiency, ASCT, and different cytogenetic abnormalities on PFS. The findings revealed that NDMM patients in DS stage III, without ASCT, in conjunction with +1q21, IgH cytogenetic abnormality, P53 deletion, or RB/D13S319 deletion, exhibited significantly poorer PFS outcomes (*p* < 0.05) as depicted in Table 3. Subsequent multivariate analysis identified without ASCT, P53 deletion, DS‐III, and +1q21 as independent prognostic factors. In contrast, IgH cytogenetic abnormality was not identified as an independent prognostic factor. In terms of OS results for NDMM patients, those with ISS‐III, DS‐III, high‐LDH, and without ASCT exhibited inferior outcomes in terms of OS (*p* < 0.05), as illustrated in Table 3. Further multivariate analysis indicated that several independent prognostic factors were identified, including without ASCT, P53 deletion, and high‐LDH, but IgH cytogenetic abnormality was not (Table 3).

**TABLE 3:** Univariate and multivariate outcome of PFS and OS analysis in NDMM.

Univariate analysis					Multivariate analysis					
	PFS		OS		PFS OS					
	*p*	HR (95% CI)	*p*	HR (95% CI)	*β*	*p*	HR (95% CI)	*β*	*p*	HR (95% CI)
IgH cytogenetic abnormality	< 0.001	1.677 (1.243–2.262)	0.010	1.681 (1.096–2.580)	0.120	0.445	1.127 (0.829–1.532)	0.230	0.308	1.259 (0.808–1.961)
No‐ASCT	< 0.001	2.179 (1.601–2.965)	< 0.001	5.691 (3.570–9.073)	0.896	< 0.001	2.450 (1.646–3.646)	1.769	< 0.001	5.865 (2.373–14.496)
1q21(+)	< 0.001	2.113 (1.596–2.797)	0.122	1.362 (0.911–2.037)	0.616	< 0.001	1.851 (1.375–2.491)	—	—	—
P53 deletion	0.004	1.784 (1.072–2.969)	0.003	2.202 (1.068–4.543)	0.550	0.009	1.734 (1.149–2.617)	0.811	0.004	2.251 (1.298–3.904)
RB(+)/D13S319(+)	< 0.001	1.746 (1.285–2.373)	0.005	1.761 (1.135–2.734)	0.226	0.147	1.254 (0.924–1.702)	0.306	0.170	1.358 (0.878–2.101)
DS‐III	< 0.001	1.879 (1.417–2.490)	0.004	2.034 (1.342–3.083)	0.339	0.049	1.403 (1.001–1.966)	0.366	0.188	1.443 (0.836–2.491)
High‐LDH	0.005	1.535 (1.099–2.143)	0.010	1.728 (1.071–2.788)	0.390	0.012	1.477 (1.088–2.004)	0.432	0.049	1.540 (1.001–2.368)
ISS‐III	0.053	1.309 (0.986–1.738)	0.003	1.806 (1.192–2.735)	—	—	—	0.262	0.224	1.300 (0.852–1.983)
Complex‐Karyotype abnormalities	0.258	1.283 (0.794–2.074)	0.059	1.740 (0.841–3.602)	—	—	—	—	—	—
Age ≥ 65 years	0.142	1.229 (0.926–1.632)	0.699	1.084 (0.718–1.635)	—	—	—	—	—	—
EMD	0.199	1.245 (0.870–1.781)	0.169	1.391 (0.824–2.350)	—	—	—	—	—	—
DS‐B	0.236	1.188 (0.885–1.594)	0.193	1.313 (0.852–2.023)	—	—	—	—	—	—

Abbreviations: ASCT, autologous hematopoietic stem cell transplantation; DS, Durie–Salmon staging system; EMD, extramedullary disease; ISS, international staging system; LDH, lactic dehydrogenase; OS, overall survival; PFS, progression‐free survival; RB(+)/D13S319(+), the absence of RB and the absence of D13S319 exist together.

### 3.7 IgH Cytogenetic Abnormality Worsens Survival of MM, Especially When Combined With +1q21, P53 Deletion, RB/D13S319 Deletion, and Increased LDH Level

**FIGURE 5:**
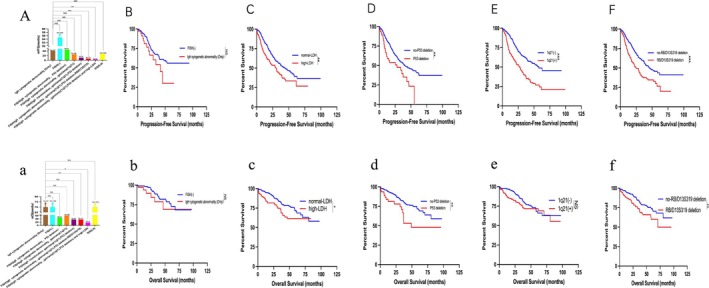
Impact of IgH cytogenetic abnormality, additional genetic abnormalities, and high‐LDH on PFS and OS in MM. (A) Kaplan–Meier estimates of PFS different strikes. (B) Kaplan–Meier estimates of PFS with or without only IgH cytogenetic abnormality. (C) Kaplan–Meier estimates of PFS with or without high‐LDH. (D) Kaplan–Meier estimates of PFS with or without P53 deletion. (E) Kaplan–Meier estimates of PFS with or without 1q21(+). (F) Kaplan–Meier estimates of PFS with or without RB/D13S319 deletion. (a) Kaplan–Meier estimates of OS different strikes. (b) Kaplan–Meier estimates of OS with or without only IgH cytogenetic abnormality. (c) Kaplan–Meier estimates of OS with or without high‐LDH. (d) Kaplan–Meier estimates of OS with or without P53 deletion. (e) Kaplan–Meier estimates of OS with or without 1q21(+). (f) Kaplan–Meier estimates of OS with or without RB/D13S319 deletion. ***p < 0.001, **p < 0.01, *p < 0.05, by two‐sided log‐rank test.

In comparison to a cohort of 194 patients with NDMM who exhibited unlabeled risk and risk factors, the isolated IgH cytogenetic abnormality group consisting of 37 NDMM patients demonstrated a trend toward shorter survival; although this trend did not achieve statistical significance. The median mPFS for the isolated IgH cytogenetic abnormality group was 41.20 months, compared to an unspecified number of months for the unlabeled risk group (*p* = 0.085, HR = 1.686 95% CI: 0.818–3.474) (Figure 5A). Additionally, the median OS for the isolated IgH cytogenetic abnormality group was not reached, compared to an unspecified number of months for the unlabeled risk group (*p* = 0.365, HR = 1.500, 95% CI: 0.542–4.149) (Figure 5a). Upon comparing the RISS‐III stage with the independent IgH cytogenetic abnormality group, no statistically significant difference was observed in PFS (28.37 vs. 41.20 months), with a *p* value of 0.248 (HR = 1.430 95% CI: 0.817–2.501) as depicted in Figure 5A. Likewise, there was no significant disparity in OS between the two groups (NR vs. NR), with a *p* value of 0.387 (HR = 1.480, 95% CI: 0.661–3.314) as illustrated in Figure 5a.

In comparison to the cohort exhibiting solely IgH cytogenetic abnormality, the cohort displaying independent IgH cytogenetic abnormality along with +1q21 and P53 deletion (triple‐hit) demonstrated a notable disparity in PFS (11.60 vs. 41.2 months); despite the absence of statistical significance between the two groups (*p* = 0.176, HR = 2.117, 95% CI: 0.508–8.830) (Figure 5A). Similarly, the observed OS of the triple‐hit group was significantly shorter than that of the IgH cytogenetic abnormality group (36.80 vs. NR); although no statistical distinction was evident between the two groups (*p* = 0.148, HR = 2.667, 95% CI: 0.445–16.010) (Figure 5a).

In comparison to the cohort with isolated IgH cytogenetic abnormality, the PFS was notably reduced in the group with combined IgH cytogenetic abnormality, +1q21, RB/D13S319 deletion, and P53 deletion (7.33 vs. 41.20 months), with a statistically significant difference (*p* < 0.001, HR = 4.117, 95% CI: 1.154–14.690) as shown in Figure 5A. Similarly, the OS was significantly shorter in the group with combined IgH cytogenetic abnormality, +1q21, RB/D13S319 deletion, and P53 deletion compared to the isolated IgH cytogenetic abnormality group (37.00 vs. NR months), with a statistically significant difference (*p* = 0.006, HR = 4.441, 95% CI: 0.879–22.440) as depicted in Figure 5a. Further, relative to the isolated IgH cytogenetic abnormality group, combined +1q21, P53 deletion, RB/D13S319 deletion, and high‐LDH had a shorter PFS (3.87 vs. 41.20 months), *p* = 0.005 (HR = 4.913 [0.461–52.410]) (Figure 5A). In addition, OS was significantly shorter in the IgH cytogenetic abnormality combined with +1q21, P53 deletions, RB/D13S319 deletion, and high‐LDH group compared to the independent IgH cytogenetic abnormality group (17.64 vs. NR months;, *p* = 0.026, HR = 5.052, 95% CI: 0.285–89.620) (Figure 5a).

## 4 Discussion

In the current investigation, it was observed that the abnormal sensitivity of MM cells, specifically identified through IgH‐related abnormalities using FISH, was markedly higher compared to detection via chromosome culture, consistent with previous research findings [11, 12]. Due to the variance in methodology between conventional karyotype chromosome analysis and FISH, the detection rate of these specific IgH‐related abnormalities is approximately tenfold higher with FISH compared to chromosome culture. Presently, FISH employs plasma cell enrichment for detection, resulting in a detection rate exceeding 100 times that of chromosome culture, indicating that the latter cannot serve as a substitute for FISH analysis [13]. A key feature of MM is the clustering of cancerous plasma cells in the bone marrow [13]. It is recommended to perform FISH upon diagnosis to identify specific cytogenetic abnormalities. As a result of the low proliferation rate of plasma cells in MM, FISH is considered more suitable than metaphase cytogenetic methods for detecting cytogenetic abnormalities in clonal cells [14]. FISH remains the preferred and efficient technique for evaluating copy number variations in tumor specimens. This is because of its straightforwardness and dependability in crucial biomarker research, establishing it as the gold standard [15, 16]. Currently, there is inconsistency in the IgH cytogenetic abnormality thresholds determined by FISH across different research centers. The ICARIA‐MM [17] and IKEMA [18] studies have established a threshold of 30% for t(14;16) and t(4;14) translocations. However, the APOLLO [19], CANDOR [20], and other studies [12] have not reported specific thresholds for these translocations. In our center, the thresholds for detecting t(4;14) and t(14;16) are set at 2.94% and 3.5%, respectively, which are lower compared to those reported in other studies. This difference may be due to variations in experimental probes and methodologies, as well as the need for larger sample sizes and external validation.

In the present study, additional analysis was conducted on individuals within the RISS‐II cohort, revealing that patients exhibiting IgH cytogenetic abnormalities experienced a shorter PFS in comparison to those lacking such cytogenetic abnormalities, thereby identifying a distinct subgroup of patients with IgH cytogenetic abnormalities. As a result of the variability in median array results within the RISS, more than half of the patients in this stage continue to be categorized in the same group. This study demonstrates a parallel between the RISS‐II stage and the classification of over 50% of NDMM patients [21, 22]. Prior research has demonstrated variations in prognosis among patients within the RISS‐II cohort, with Mayo MASS and R2ISS staging systems offering improved categorization [23, 24]. Previous research has indicated that individuals classified as RISS‐II patients with high‐risk cytogenetic abnormalities (HRCA) or a singular type of HRCA exhibit decreased median PFS, OS, and duration of response (DOR) in comparison to patients lacking HRCA (*p* < 0.05). Conversely, the time to response (TTR) is notably extended (*p* < 0.05) in these subgroups [25].

Consistent with prior findings, ASCT demonstrates the ability to mitigate the prognostic impact of IgH cytogenetic abnormality. Notably, ASCT yields significant enhancements in PFS and OS among patients with myeloma exhibiting IgH cytogenetic abnormality. Based on multiple studies, ASCT has been shown to mitigate the negative prognosis of patients with the t(11;14) chromosomal abnormality, resulting in a higher survival rate for transplanted African American individuals compared to their White counterparts in the United States [26]. Combining Bortezomib with thalidomide and dexamethasone (VTD), followed by double ASCT, has shown promise in mitigating the unfavorable prognosis linked to t(4;14) [27]. However, the impact of Bortezomib maintenance therapy on prognosis compared to thalidomide maintenance therapy is minimal. Additional studies have demonstrated that ASCT has the potential to enhance treatment efficacy and prolong life expectancy, particularly in high‐risk patient populations, and may serve as a valuable tool for refining risk stratification in subsequent research [28].

This study focused on investigating the clinical characteristics, outcomes, and prognosis of NDMM with IgH cytogenetic abnormalities. The study found that patients with IgH cytogenetic abnormalities had unfavorable prognostic factors, including advanced disease stage (DS, ISS, RISS, and R2ISS). IgH cytogenetic abnormalities are regarded as primary events in the development of MM [29]. Additionally, the presence of IgH cytogenetic abnormalities was associated with genetic abnormalities such as del(13q14), del(17p), and +1q21, leading to poorer PFS and OS. However, IgH cytogenetic abnormalities alone do not serve as independent prognostic factors. In the standard crisis group, t(6;14) and t(11;14) are observed, while in the high‐risk group, t(4;20), t(14;14), and t(14;16) are present, as reported by mSMART 3.0 [30]. In recent years, multiple retrospective analyses and prospective studies have shown that patients with t(11;14) have a similar or even better prognosis than those without t(11;14) [31]. Nevertheless, recent studies suggest that t(11;14) may indicate intermediate risk and is linked to shorter survival in the context of novel agents [32]. A study found that 12% of NDMM had t(11;14). In univariate analysis, there was no significant difference in first‐line PFS between patients with t(11;14) and those without it (*p* = 0.567, HR = 1.07, 95% CI: 0.72–1.2), but there was a trend toward worse OS (*p* = 0.042, HR = 1.36, 95% CI: 1.01–1.8) [33]. A retrospective study pointed out that MM patients carrying t(11;14) without other high‐risk abnormalities had a significantly shorter median PFS compared to patients without t(11;14) and without high‐risk features (36.1 vs. 40.1 months, *p* = 0.028) [34]. However, in our study cohort, only 6.5% of NDMM had t(11;14). No significant differences in PFS and OS were observed between patients with t(11;14) and those without IgH cytogenetic abnormalities. This discrepancy could result from the small sample size and the need for multicenter validation. Additionally, t(14;16) is classified as a high‐risk factor in m‐SMART risk stratification and has prognostic implications consistent with prior research [35, 36]. Moreover, it has been observed that around 13.17% of cases of multiple myeloma display disruption of the IgH gene as detected by FISH, with no specific partner gene identified. The presence of the t(14; undefined) aberration has been associated with poorer survival outcomes compared to cases without IgH cytogenetic abnormalities, contrary to previous studies. To strengthen the credibility of these findings, it is advisable to enlarge the sample size for further validation [10].

IgH cytogenetic abnormalities significantly impact NDMM patient prognosis. In a recent study of patients with NDMM, it was found that approximately 25% of patients exhibited one high‐risk abnormality, while about 3% of patients exhibited two high‐risk abnormalities [37]. Consistent with the aforementioned results, we identified a single high‐risk abnormality in 37 out of 503 patients. Our study revealed a PFS of 41.20 months for patients with a single high‐risk abnormality, compared to 11.60 months for patients with triple‐hit multiple myeloma. Additionally, the OS for patients with a single high‐risk abnormality was not reached, while patients with triple‐hit multiple myeloma had an OS of 36.8 months. In this perspective, although no statistically significant difference was seen between the double‐hit, triple‐hit had significantly shorter PFS than patients with only IgH cytogenetic abnormalities. These may be related to the sample size; it was found through calculation that the sample size of both groups needs to be increased to 50 cases. In a case report, a patient with rapid progression and poor prognosis was identified as having double‐hit multiple myeloma, characterized by both IgH/CCND1 and IgH/MYC translocations [38]. A comparable case study presented a patient with double‐hit plasma cell leukemia characterized by IgH/BCL2 and IgH/MYC translocations [39]. It was subsequently determined that individuals with IgH cytogenetic abnormality, +1q21, P53 deletion, and RB1/D13S319 deletion exhibited significantly shorter OS and PFS compared to those with only IgH cytogenetic abnormality.

The innovation of this study lies in its detailed analysis of specific IgH translocations, such as t(14;16) and t(14; undefined), and their correlation with poor prognosis, as well as the demonstration that ASCT can improve outcomes in patients with IgH cytogenetic abnormalities. This study also encountered several limitations. Firstly, this study was a retrospective cohort study, and future prospective studies with larger, more diverse cohorts, potentially utilizing multicenter collaborations or public databases, could offer more comprehensive and generalizable conclusions. Secondly, in nearly one‐third of cases, the specific sites of IgH cytogenetic abnormality were not detected, and the detection rate of IgH cytogenetic abnormality could be improved with more refined techniques, such as second‐generation sequencing.

## 5 Conclusion

Due to the sluggish rate of plasma cell proliferation in MM, the FISH method is regarded as a viable approach for identifying clonal cell cytogenetic abnormalities. The prognostic implications of double‐ or triple‐hit MM warrant further elucidation and characterization, as this information will inform personalized treatment strategies for MM patients.

## Author Contributions

**Yanqiu Xiong:** writing – original draft, data curation, investigation, formal analysis. **Meng Li:** writing – original draft, data curation, investigation, formal analysis. **Yan Li:** data curation. **Wenjiao Tang:** data curation. **Tian Dong:** data curation. **Bing Xiang:** data curation. **Li Zhang:** writing – review and editing, funding acquisition, methodology, validation, project administration, supervision. **Ling Pan:** writing – review and editing. **Ting Niu:** writing – review and editing.

## Conflicts of Interest

The authors declare no conflicts of interest.

## Data Availability

The data that support the findings of this study are available on request from the corresponding author. The data are not publicly available due to privacy or ethical restrictions.
